# Fate of Nutrients in Shallow Groundwater Receiving Treated Septage, Malibu, CA

**DOI:** 10.1111/gwat.12194

**Published:** 2014-06-05

**Authors:** John A Izbicki

**Affiliations:** Water Resources, U.S. Geological Survey4165 Spruance Road, Suite 200, San Diego, CA 92101; (619) 225-6131; fax: (619) 225-6101

## Abstract

Treated wastewater discharged from more than 400 onsite wastewater treatment systems (OWTS) near the Civic Center area of Malibu, California, 40 km west of downtown Los Angeles, composes 28% of the recharge to a 3.4 km^2^ alluvial aquifer. On the basis of δ^18^O and δD data, the fraction of wastewater in some samples was >70%. Ammonium and nitrate concentrations in water from 15 water-table wells sampled in July 2009 and April 2010 ranged from <0.01 to 12 milligrams per liter as nitrogen (mg/L as N), and from <0.01 to 11 mg/L as N, respectively. Chemical and isotopic data (δ^15^N of ammonium and nitrate, and δ^18^O of nitrate) show two processes remove nitrogen discharged from OWTS. Where groundwater was reducing, sorption of ammonium resulted in 30 to 50% nitrogen removal. Where groundwater was initially oxic, nitrification with subsequent denitrification as reducing conditions developed, resulted in up to 60% nitrogen removal. Nitrogen removal through sorption dominated during the cooler April sample period, and denitrification dominated during the warmer July sample period. The combination of mixing and nitrogen removal due to denitrification, sorption, and volatilization produces a δ^15^N apparent fractionation factor (ε_app_ = −5), that can be explained using laboratory-derived fractionation factors (ε) for the individual processes. Phosphate concentrations ranged from < 0.04 to 2 mg/L as phosphorous. Sorption to iron oxides on the surfaces of mineral grains at near-neutral pH's removed some phosphate; however, little removal occurred at more alkaline pH's (>7.3).

## Introduction

**Figure 1:**
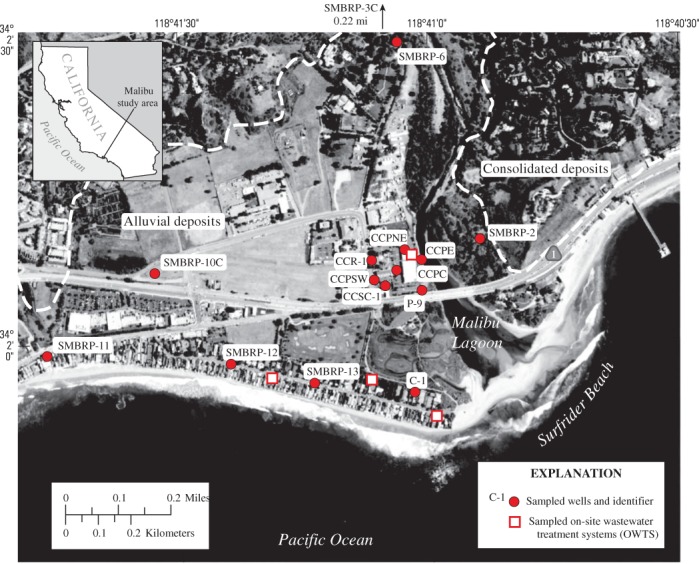
Study area location.

Residential and commercial wastewater is treated in onsite wastewater treatment systems (OWTS) and discharged to groundwater in the Civic Center area of Malibu, California, 40 km west of downtown Los Angeles (Figure 1). More than 400 OWTS are present in and near the Civic Center area, and discharges from OWTS compose 28% of the recharge to the 3.4 km^2^ alluvial aquifer underlying the area (Stone Environmental, Inc. 2010). Groundwater containing treated wastewater discharges to environmentally sensitive receiving waters in Malibu Lagoon, and to nearby recreational beaches. Local regulatory agencies believe discharging groundwater may be a source of nutrients and fecal indicator bacteria (FIB) to Malibu Lagoon and nearby recreational beaches. In 2009 these agencies banned additional unsewered residential and commercial development in the area, and mandated construction of a sewer and wastewater treatment system (RWQCB 2009).

Recent work showed groundwater underlying the Civic Center area does not contain high concentrations of FIB from OWTS discharges (Izbicki et al. 2011, 2012a); and is not the source of high FIB in the lagoon and near-shore ocean. However, several studies have suggested that groundwater containing high concentrations of nitrogen from OWTS discharges is the largest source of nutrients to Malibu Lagoon (Ambrose and Orme 2000; Suffet and Sheehan 2000; Stone Environmental, Inc. 2004; Sutula et al. 2004). Collectively, these studies recognize that nitrate removal occurs within groundwater, and not all the nitrate discharged by OWTS reaches the lagoon or near-shore ocean. However, these studies did not provide a quantitative, process-oriented assessment of the fate of nutrients within groundwater.

### Chemical and Microbiological Processes Associated with Nitrogen

Adult humans excrete between 2.8 and 3.7 kg of N per year per capita (Follett and Follett 2008). Ammonium and organic nitrogen are present in OWTS discharges at concentrations ranging from 25 to 60 mg/L as N, and although concentrations range widely, typical values are about 40 to 45 mg/L as N (Umari et al. 1993; Wakida and Lerner 2005; Katz et al. 2010). After discharge from OWTS, some ammonium and organic nitrogen may be lost through assimilation into bacteria, sorption to clay minerals, or depending on pH, nitrogen in the form of ammonium may be lost through volatilization. Ammonium and organic nitrogen discharged from OWTS may be oxidized to nitrate in a series of bacterially mediated steps: 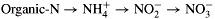


The portion of the reaction describing the conversion of ammonium to nitrate is commonly referred to as nitrification: 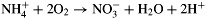


Depending on the microorganisms involved and environmental factors such as pH and temperature, various nitrogen oxides and intermediate products (NO_2_^−^, NO, N_2_O) may be produced (Paul and Clark 1996). About two-thirds of the oxygen incorporated into nitrate during nitrification is derived from hydrolysis of water and only one-third is derived from oxygen gas (Mayer et al. 2001).

Nitrate reduction and denitrification are the dominant nitrate removal processes in anaerobic groundwater. Nitrate reduction results in the chemical reduction of nitrate to various oxides of nitrogen. Denitrification results in the conversion of nitrate to nitrogen gas and removal of nitrogen from the aquifer system: 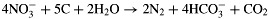


Nitrate reducing and denitrifying bacteria are ubiquitous, and the presence of oxygen or other electron donors are the primary limiting factors on the rate of denitrification (Paul and Clark 1996; Rivett et al. 2008). Other environmental factors such as nitrate concentration, pH, temperature, and the presence of labile (reactive) organic carbon exert a secondary influence on the rate of denitrification (Rivett et al. 2008).

### Nitrogen and Oxygen Isotope Systematics

The combination of chemical and isotopic data can be used to understand environmental processes controlling nitrate concentrations in groundwater (Choi et al. 2003). In many areas, interpretation of isotopic data is complicated by the wide, often overlapping range in concentration and nitrogen isotopic composition of nitrate from different sources, and by the changing isotopic composition of nitrate as reactions proceed (Delwiche and Steyn 1970; Mariotti et al. 1981; Xue et al. 2009). Sources and processes affecting nitrate concentrations can be evaluated through the dual use of the nitrogen isotopes of ammonium and nitrate, and the oxygen isotopes of nitrate (Durka et al. 1994; Kendall 1998; Choi et al. 2003).

#### Nitrogen Isotopes of Ammonium and Nitrate

There are two stable isotopes of nitrogen: nitrogen-14 (^14^N) and nitrogen-15 (^15^N). The average ^15^N/^14^N ratio in atmospheric air (1/272) is constant, and nitrogen isotope values are reported using standard delta notation (δ) in per mil (parts per thousand) differences relative to the ^15^N/^14^N ratio of nitrogen gas in atmospheric air. Using this notation, the atmospheric standard has a δ^15^N value of 0 per mil. Positive δ^15^N values contain more of the heavier isotope than atmospheric nitrogen gas, and negative δ^15^N values contain less of the heavier isotope. The biological reactivity and wide range of oxidation states in nitrogen compounds results in a wide range in δ^15^N isotopic compositions spanning more than 200 per mil relative to standard atmospheric nitrogen gas (Coplen et al. 2001). The δ^15^N composition of ammonium and nitrate are commonly written as δ^15^N-NH_4_ and δ^15^N-NO_3_, respectively.

The separation of heavier and lighter isotopes during physical, chemical, or biological reactions is known as fractionation. In general, the lighter isotope is preferentially incorporated into reaction products while the heavier isotope preferentially remains in the reactant. Isotopic fractionation can be modeled as a Rayleigh process using the following equation: 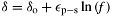
where δ is the isotopic composition of the product, p, in per mil, δ_o_ is the initial isotopic composition of the substrate (reactant), s, in per mil, ε_p-s_ is the isotopic enrichment factor, in per mil, and *f* is the remaining fraction of the substrate, unit less.

The isotopic enrichment factor is defined as: 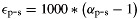
where α_p-s_ is fractionation factor defined as the ^15^N/^14^N ratio of the product (p) divided by the ^15^N/^14^N of the substrate (s).

The δ^15^N-NH_4_ composition in OWTS discharges is relatively constant, averaging 4.9 ± 0.4 per mil (Hinkle et al. 2008; Katz et al. 2010). Ammonium containing the lighter isotope would be preferentially lost during volatilization of ammonia, with a fractionation factor of ε = −13 to −38 (Delwiche and Steyn 1970; Mariotti et al. 1981; Casciotti et al. 2003). As volatilization continues the remaining ammonium would be isotopically heavier than ammonium in OWTS discharges. In contrast, there is little isotopic fractionation of nitrogen in ammonium during sorption, ε = 1, with a slight preference for sorption of ammonium containing the heavier isotope, leaving similar or slightly lighter nitrogen in the remaining ammonium (Delwiche and Steyn 1970; Karamanos and Rennie 1978).

The δ^15^N-NO_3_ composition in OWTS discharges varies more widely than δ^15^N-NH_4_ compositions, averaging 7.2 ± 2.6 per mil (Hinkle et al. 2008). Nitrification of ammonium to nitrate would be expected to result in lighter δ^15^N-NO3 values in the product (nitrate). However, δ^15^N-NO_3_ values from septic sources are commonly heavier than δ^15^N-NH_4_ in septic discharges (Hinkle et al. 2008). This may occur as a result of volatilization of ammonium prior to nitrification or from losses of nitrogen oxide intermediates formed during nitrification, with subsequent oxidization of the remaining isotopically heavier nitrogen to nitrate. Heavier δ^15^N-NO_3_ values from septic sources also can result from denitrification and the subsequent loss of isotopically light nitrogen gas. Regardless of the process, the heavier δ^15^N-NO_3_ from septic sources compared to the δ^15^N-NH_4_ is not random and represent a loss of nitrogen during nitrification.

Denitrification causes the δ^15^N-NO_3_ values to increase as nitrate concentrations decrease. As previously discussed, isotopic fractionation during denitrification can be modeled as a Raleigh process. Fractionation factors for denitrification estimated from field data (apparent fractionation factors, ε_app_) range from −40 to −5 per mil (Kendall and Aravena 2000). Fractionation factors estimated from more controlled laboratory studies (ε) vary less and range from −29 to −25 per mil (Mariotti et al. 1981).

#### Oxygen Isotopes of Nitrate

The δ^18^O composition of nitrate is commonly written as δ^18^O-NO_3_, and values in nitrate range between −10 to 80 per mil (Kendall 1998). The δ^18^O composition of nitrate from different sources varies less widely, and heavier values result from denitrification. For example, the δ^18^O-NO_3_ composition of inorganic fertilizer commonly ranges between 18 to 22 per mil, soil has δ^18^O-NO_3_ compositions between −2 and 6 per mil, and the δ^18^O-NO_3_ composition of atmospheric deposition ranges between 30 and 70 per mil (Choi et al. 2003). δ^18^O-NO_3_ can be used in conjunction with chemical and δ^15^N-NO_3_ data to identify nitrate sources and processes, such as denitrification, that affect nitrate concentrations (Aravena and Robertson 1998; Choi et al. 2003).

The δ^18^O-NO_3_ composition formed during nitrification is a function of the δ^18^O composition of water and of molecular oxygen (either atmospheric, soil gas, or dissolved in water). As previously discussed, about two-thirds of the oxygen incorporated into nitrate during nitrification is derived from hydrolysis of water and the remaining one-third is derived from molecular oxygen (Mayer et al. 2001). The oxygen isotopes of nitrate fractionate with denitrification in a similar manner as the nitrogen isotopes with the lighter isotope reacting preferentially and the heavier isotope remaining in the unreacted nitrate. Denitrification results in an enrichment of the oxygen-18 and nitrogen-15 in the remaining nitrate of about 2 to 1 (Amberger and Schmidt 1987; Bottcher et al. 1990).

#### Quantification of Isotopic Fractionation Processes

Field data (Kendall and Aravena 2000) show that apparent fractionation factors (ε_app_) commonly differ from laboratory-derived fractionation factors (ε) (Mariotti et al. 1981). The reasons for these differences are poorly understood but have been known to occur for nitrogen isotope fractionation during denitrification (Mariotti et al. 1981; Smith et al. 1991), and for a number of other isotopic systems including: carbon isotope fractionation during methyl tert-butyl ether degradation (Lesser et al. 2008), oxygen isotope fractionation during O_2_ reduction (Revesz et al. 1999), Cl and O isotope fractionation during perchlorate degradation (Hatzinger et al. 2009), selenium isotope reduction during selenium reduction (Clark and Johnson 2008), and chromium isotope fractionation during chromium reduction (Berna et al. 2009; and Izbicki et al. 2012b).

An analytical solution used to evaluate differences between ε_app_ and ε by Abe and Hunkeler (2006) shows advective and dispersive mixing resulting from aquifer heterogeneity leads to ε_app_ values that are less negative than ε, and concludes that ε_app_ will underestimate the extent of reactions occurring in environmental systems. On a regional scale, differences between ε_app_ and ε for denitrification for groundwater within alluvial deposits in the Central Valley of California (Green et al. 2010) were attributed to mixing caused by heterogeneity within the aquifer system. Although work by Abe and Hunkeler (2006) and Green et al. (2010) focused on advective and dispersive mixing within aquifers as the source of differences between ε and ε_app_, it is possible that complex biogeochemical processes operating within environmental systems also may contribute to our poor understanding of isotopic fractionation in field settings, thereby limiting quantitative assessment of reactions occurring in environmental systems.

### Chemical Processes Associated with Phosphorus

Controls on phosphorus concentrations through precipitation of phosphorus containing minerals are minimal (Hem 1985). However, phosphorus is strongly sorbed to iron and alumina oxides on solid particles (Hemwell 1957; Mueller and Helsel 1996; Zhang and Huang 2007), clay minerals (Parfitt 1978), and calcium carbonate mineral surfaces (Cole et al. 1953). Because of sorption, phosphorus transport though groundwater has long been assumed to be negligible and phosphorus transport to surface receiving waters has been focused largely on surficial and sediment transport pathways (Mueller and Helsel 1996). Recent work has shown sorption is pH dependent and that as available sorption sites approach saturation, phosphorus transport through groundwater may occur (Domagalski and Johnson 2011).

Unlike nitrogen, there are no naturally occurring isotopes of phosphorus that can be used to evaluate chemical processes affecting phosphorus concentrations. However, recent work used differences in the oxygen isotopic composition of phosphate to evaluate phosphorus sources and reactions in environmental settings (McLaughlin et al. 2006a, 2006b; Jaisi et al. 2011).

## Purpose and Scope

The purpose of this study is to evaluate nitrogen transformations and phosphate chemistry in shallow groundwater receiving large amounts of treated wastewater from OWTS discharges. Scope of the study included sample collection and analyses from existing water-table wells and from within OWTS. Chemical analysis included nitrogen (ammonium, organic nitrogen, nitrate, and nitrite) and phosphorus (total phosphorus and orthophosphorus) concentrations. Isotopic analysis included: (1) δ^18^O and δD of water to estimate the fraction of water derived from imported water having a wastewater history, and (2) δ^15^N-NH_4_ and δ^15^N-NO_3_, and δ^18^O-NO_3_ to evaluate processes affecting nitrogen concentrations and transformations.

## Hydrogeology

The climate of the Civic Center area of Malibu, about 40 km west of downtown Los Angeles, California (Figure 1), is Mediterranean, with cool wet winters and warm dry summers. Average annual precipitation, falling mostly as rain during winter storms between November and March, is 340 mm. The area contains 3.4 km^2^ of alluvium (Yerkes and Campbell 1980), having a maximum depth of approximately 60 m below sea level (Stone Environmental, Inc. 2004). Malibu Lagoon and associated wetlands occupy about 9 ha near the eastern edge of the alluvium. Alluvium near the water table consists of sand and gravel overlying a fine-grained confining layer; deposits are finer-grained and contain more organic material near Malibu Lagoon. The alluvial deposits are surrounded and underlain by low-permeability consolidated marine, nonmarine, and volcanic rock that compose the Santa Monica Mountains (Yerkes and Campbell 1980). Prior to the importation of water, the alluvial deposits were a source of public supply; however, these deposits are not presently pumped, and water from northern California and the Colorado River is imported for public supply.

Land use in the Civic Center area includes undeveloped land (including parkland surrounding Malibu Lagoon), low-density and high-density residential, and commercial uses. The area is unsewered. Residential and commercial wastewater is treated by OWTS prior to discharge to groundwater. More than 400 OWTS were in and near the Civic Center area, 49 of these systems served commercial properties (Stone Environmental, Inc. 2010). Most OWTS were conventional septic systems, however almost 30 advanced systems were in use by 2010 (Stone Environmental, Inc. 2010). The advanced systems commonly contain multiple treatment processes intended to reduce fecal bacterial and nutrient concentrations.

Groundwater recharge occurs as infiltration of streamflow from Malibu Creek, infiltration of runoff from the surrounding uplands, direct infiltration of precipitation, and as groundwater discharge from surrounding consolidated rock. As a result of development, additional recharge also occurs from infiltration of water imported for public supply that is discharged through OWTS. Recharge from OWTS discharges has been estimated to be about 1050 m^3^/d, or about 28% of the total recharge to the alluvial aquifer (McDonald Morrissey Associates, Inc. 2010). The large percentage of recharge from OWTS discharges, containing high dissolved organic carbon concentrations, may have altered redox conditions within the aquifer—increasing the potential for nitrogen removal through denitrification. With the exception of residential areas north of the lagoon, which were not sampled as part of this study, the quantity of water used for landscape irrigation and recharge from irrigation return is small.

Historically, depth to water in the alluvial deposits ranged from 0 m near the lagoon and ocean to about 10 m below ground surface in upland areas (Birkeland 1972; Orme et al. 2000; Stone Environmental, Inc. 2004). Minimum depth to water for discharge from OWTS is commonly 1 m, for areas having percolation rates 2 min/cm or greater (RWQCB 2004, 2010). During the wet season, Malibu Lagoon is open to the ocean and water levels within the lagoon and the surrounding alluvial aquifer vary with the daily tidal cycle. Median depth to water in sampled wells was 2.5 m, near the end of the wet season in April 2010. During the dry season, a berm develops at the mouth of Malibu Lagoon separating the lagoon from the ocean, and water levels in the lagoon and parts of the surrounding alluvial aquifer vary less and are higher than during the wet season. Median depth to water in sampled wells was 1.8 m, in July 2009 when the berm in the lagoon was closed.

**Figure 2:**
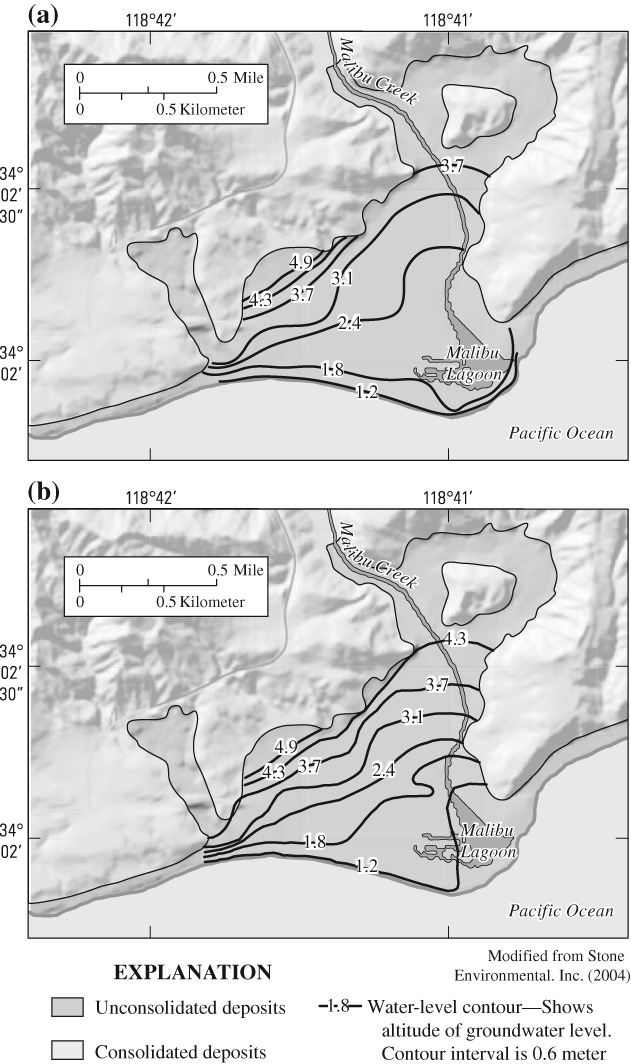
Water-table contours in alluvial deposits near the Civic Center area, Malibu, California, (a) September 2003 and (b) March 2004 (from Stone Environmental, Inc. 2004).

Discharge from the alluvial aquifer occurs to Malibu Lagoon and to the near-shore ocean (Figure 2). Although seasonal differences are important, on average about half the groundwater discharges to Malibu Lagoon, about 40% to the near-shore ocean, and the remainder discharges through evapotranspiration (McDonald Morrissey Associates, Inc. 2010).

## Methods

### Field Methods

Samples were collected from existing water-table monitoring wells installed as part of previous studies of the impact of OWTS discharges to the study area (Figure 1). Eleven wells were sampled in July 2009. Fifteen wells were sampled in April 2010. Of the four additional wells, two were located in the commercial district adjacent to Malibu Lagoon and two were located close to Malibu Creek upstream of the commercial district. Most sampled wells were screened near the water table and depths to water ranged from 1.3 to 5.6 m (Table 1). Samples also were collected from within conventional residential, advanced residential, and commercial OWTS prior to discharge to the leaching system during October 2009 and July 2010 (Figure 1).

**Table 1:** Well-Construction, Water-Level Data, and Percent Imported Water for Sampled Wells, Civic Center Area, Malibu, California

Well Name (Location Shown on Figure 1)	USGS Well Number	Well Depth (m)	Top and Bottom of Screened Interval (m)	Nearby Land Use	Date (m/dd/yyy)	Depth to Water (m)	Percent Imported Water
			Wells				
SMBRP-3c	340242118410501	9.1	4.5–9.1	Residential	4/21/2010	5.6	19
SMBRP-6	340230118410401	7.6	3.0–7.6	Residential	4/17/2010	4.5	9
SMBRP-11	340159118414601	6.1	1.5–6.1	Residential	7/21/2009	2.6	76
SMBRP-11	340159118414601	6.1	1.5–6.1	Residential	4/21/2010	2.5	46
SMBRP-12	340158118412401	7.6	3.0–7.6	Residential	7/22/2009	2.1	60
SMBRP-12	340158118412401	7.6	3.0–7.6	Residential	4/21/2010	2.1	75
SMBRP-13	340156118411401	6.1	1.5–6.1	Residential	7/22/2009	2.3	39
SMBRP-13	340156118411401	6.1	1.5–6.1	Residential	4/20/2010	2.5	23
C-1	340155118410201	4.4	1.2–4.4	Commercial	7/26/2009	1.4	22
C-1	340155118410201	4.4	1.2–4.4	Commercial	4/19/2010	2.0	50
CCPC	340207118410401	6.9	—	Commercial	7/23/2009	1.8	22
CCPC	340207118410401	6.9	—	Commercial	4/18/2010	2.5	19
CCPE	340208118410101	16.1	3.7–15.8	Commercial	7/23/2009	1.5	17
CCPE	340208118410101	16.1	3.7–15.8	Commercial	4/17/2010	2.7	53
CCPNE	340209118410301	7.6	—	Commercial	7/23/2009	1.8	23
CCPNE	340209118410301	7.6	—	Commercial	4/18/2010	2.8	26
CCPSW	340206118410701	6.2	—	Commercial	4/18/2010	2.6	27
CCR-1	340208118410701	5.9	—	Commercial	7/24/2009	1.7	19
CCR-1	340208118410701	5.9	—	Commercial	4/18/2010	2.4	21
CCSC-1	340203118410701	4.5	—	Commercial	4/18/2010	2.2	71
P-9	340205118410101	4.2	1.3–4.2	Commercial	7/22/2009	1.3	30
P-9	340205118410101	4.2	1.3–4.2	Commercial	4/18/2010	2.6	69
SMBRP-10C	340207118413301	7.6	3.0–7.6	Undeveloped	7/21/2009	1.9	3
SMBRP-10C	340207118413301	7.6	3.0–7.6	Undeveloped	4/17/2010	1.3	1
SMBRP-2	340210118405401	7.6	3.0–7.6	Undeveloped	7/22/2009	1.6	13
SMBRP-2	340210118405401	7.6	3.0–7.6	Undeveloped	4/19/2010	2.7	3
			Onsite wastewater treatment systems				
Conventional	340156118410701	—	—	Residential	10/1/2009	—	100
Conventional	340156118411101	—	—	Residential	7/7/2010	—	100
Advanced	340152118405901	—	—	residential	10/1/2009	—	100
Advanced	340156118412001	—	—	Residential	7/7/2010	—	100
Commercial	340208118411001	—	—	Commercial	7/7/2010	—	100

Samples for nitrogen and phosphorus species were filtered through 0.45 µm pore-sized filters at the time of collection, chilled on ice, and shipped within 24 hours to the U.S. Geological Survey's National Water Quality Laboratory (NWQL) in Denver, Colo. for analysis. Samples intended for analysis of δ^18^O and δD in water were unfiltered and shipped to the Reston Stable Isotope Laboratory (RSIL) in Reston, Va. Samples intended for analysis of δ^15^N-NH_4_, δ^15^N-NO_3_, and δ^18^O-NO_3_ were held until completion of NH_4_ and NO_3_ analysis and then shipped with that information to the RSIL for analysis.

### Laboratory Methods

#### Chemical Analyses

Water samples were analyzed by the U.S. Geological Survey's National Water Quality Laboratory (NWQL) in Denver, Colorado. Ammonium, nitrate plus nitrite, nitrite, and orthophosphate were determined by automated colorimetery (Fishman 1993). Ammonia plus organic nitrogen and total phosphorus were determined by Kjeldahl digestion (Patton and Truitt 1992, 2000; Patton and Truitt 2000). Major-ions, selected minor, and selected trace elements were analyzed using methods described by Fishman and Friedman (1989), Fishman (1993), and Garbarino et al. (2002, 2006). Not all these data are discussed in this paper; however, data are available online from the U.S. Geological Survey's computerized data base National Water Information System (NWIS) at http://waterdata.usgs.gov/nwis.

#### Isotopic Analyses

Water samples were analyzed for isotopes including: (1) δ^18^O and δD of water and (2) δ^15^N-NH_4_ and δ^15^N-NO_3_, and δ^18^O-NO_3_.

Samples for analysis of δ^18^O and δD in water were analyzed by mass spectrometry using methods by Epstein and Mayeda (1953) and Coplen et al. (1991), respectively. Results are reported in delta notation (δ) as per mil (‰) differences relative to Vienna standard mean ocean water (VSMOW) according to the following: 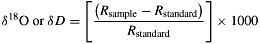
where *R*_sample_ and *R*_standard_ refer to the ratio in the sample and the standard, respectively. Values were normalized on scales such that the oxygen and hydrogen isotopic values of standard light Antarctic precipitation (SLAP) are −55.5 per mil and −428 per mil, respectively (Gonfiantini 1978, 1984; Hut 1987; Coplen 1988, 1994).

Samples for δ^15^N-NH_4_ were measured by continuous-flow isotope-ratio mass spectrometry (CF-IRMS) (Hannon and Bohlke 2008). δ^15^N-NO_3_ and δ^18^O-NO_3_ were analyzed by CF-IRMS (Révész and Casciotti 2007) using a culture of denitrifying bacteria, *Pseudomonas aureofaciens*, for the enzymatic conversion of NO_3_^−^ to N_2_O prior to analyses (Sigman et al. 2001). The ^17^O isotopic abundance was accounted for prior to reporting δ^15^N-NO_3_ values. For samples containing atmospheric nitrate, the bacterial method may overestimate δ^15^N values by as much as 1 to 2 per mil. δ^15^N-NH_4_ and δ^15^N-NO_3_ were reported in delta notation as per mil differences relative to the standard atmospheric nitrogen composition which has a value of 0 per mil. δ^18^O-NO_3_ was reported in delta notation as per mil differences relative to VSMOW.

δ^15^N-(NH_4_ + NO_3_), were calculated from δ^15^N-NH_4_ and δ^15^N-NO_3_ compositions according to the following: 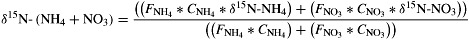

where *F* is the fraction of ammonium (*F*_NH4_) or nitrate (*F*_NO3_) composing the total nitrogen composition. For example, *F*_NH4_ = (*C*_NH4_)/(*C*_NH4_ + *C*_NO3_), neglecting organic nitrogen concentrations which are small, and *C* in the concentration of ammonium (*C*_NH4_) or nitrate (*C*_NO3_).

For samples having ammonium or nitrate concentrations too low to permit independent analysis of δ^15^N-NH_4_ and δ^15^N-NO_3_ the isotopic composition of the dominant form was assumed to represent the δ^15^N-(NH_4_ + NO_3_) composition.

## Results

On the basis of 26 samples collected from 15 wells in July 2009 and April 2010, water from sampled wells in the alluvial aquifer underlying the Civic Center area of Malibu, California had a median specific conductance of 2340 µS/cm. Water from some wells near Malibu Lagoon (C-1, CCPE, and SMBRP-13), and near wetlands along the western margin of the Civic Center area, (SMBRP-10C) had specific conductances ranging from about 8000 to 26,000 µS/cm. Dissolved oxygen concentrations in water from sampled wells ranged from less than the detection limit of 0.2 to 5.8 mg/L, with a median concentration of 0.5 mg/L (Table S1).

Nitrate concentrations in water from sampled wells ranged from less than the reporting limit of 0.01 to 11 mg/L as N (Table S1), with a median concentration of 1.1 mg/L as N. Nitrate was the primary form of nitrogen in 15 of 26 samples. Water from only one well exceeded the U.S. Environmental Protection Agency Maximum Contaminant Level for drinking water of 10 mg/L as N. Nitrite concentrations were generally less than the reporting limit of 0.001 mg/L as N, although water from one well had nitrite concentrations as high as 0.027 mg/L as N (Table S1).

Ammonium concentrations in water from sampled wells ranged from less than the reporting limit of 0.01 to 12 mg/L as N (Table S1), with a median concentration of 0.04 mg/L as N. Ammonium was the primary form of nitrogen in 6 of 26 samples. Ammonium plus organic nitrogen concentrations were similar, consistent with only small amount of organic nitrogen present in water from sampled wells. Although not directly applicable for groundwater samples, water from 32% of samples exceeded the U.S. Environmental Protection Agency (1989) acute criterion for marine aquatic life of 0.46 mg/L as N, and all samples exceeded the chronic criterion for marine aquatic life of 0.035 mg/L as N.

Phosphorus concentrations in water from sampled wells ranged from less than the reporting limit of 0.04 to 2 mg/L as phosphorus (P), with a median concentration of 0.28 mg/L as P. Almost all the phosphorus was in the form of orthophosphate. Although not directly applicable for groundwater samples, water from 85% of samples exceeded the U.S. Environmental Protection Agency (1989, 2009) criterion for phosphorus in marine and estuarine water of 0.1 mg/L, and 27% of samples exceeded suggested criteria of 0.71 mg/L to limit stream eutrophication (Dodds et al. 1998; Dodds 2006a, 2006b).

Consistent with the results of previous studies in other areas (Katz et al. 2010), nitrogen and phosphorus concentrations ranged widely in water within sampled OWTS during the study. In general, ammonium was the primary form of nitrogen with concentrations in residential OWTS ranging from 3.1 to 43 mg/L as N (Table S1). The higher value is more typical of average ammonium concentrations in residential OWTS (Umari et al. 1993; Wakida and Lerner 2005; Katz et al. 2010), and is consistent with concentrations used for calculations in this paper estimated from literature values. The ammonium concentration in water within the sampled commercial OWTS was 340 mg/L as N (Table S1). This OWTS serves a restaurant, and the high concentration may result from the lack of dilution by gray water (from sources such as laundry and shower water), or from the wastes associated with food preparation. Nitrate concentrations in water within sampled OWTS were generally low, with the exception of one sample from a conventional residential OWTS having a nitrate concentration of 24 mg/L as N (Table S1). Phosphorus concentrations in water within sampled residential OWTS ranged from 0.82 to 7.6 mg/L as P (Table S1). Similar to ammonium, lack of dilution by gray water or addition of food wastes may have resulted in the higher phosphorus concentrations in the sampled commercial OWTS of 12 mg/L as P (Table S1).

The δ^18^O and δD composition of water from sampled wells ranged from −3.7 to −8.6, and −29 to −65 per mil, respectively. δ^18^O and δD values within sampled OWTS were lighter (more negative), and ranged from −9.1 to −9.6 and −72 to −77 per mil, respectively (Table S2). These values are more negative than the δ^18^O and δD composition of precipitation along the California coast near Santa Maria, California about 200 km northwest of the study area (IAEA 1992), and more negative than the δ^18^O and δD composition of water from Malibu Creek collected as part of this study in April 2010 (−4.6 and −32 per mil, respectively).

The percent imported water, having a wastewater history in a given sample (Table 1), was calculated as a two-part mixture of native water (estimated from the δD composition of Malibu Creek, −32 per mil) and the average δD composition of water from sampled OSWT systems, −74 per mil. The percentage of imported water from wells SMBPR-13, C-1, and CCPE, having a specific conductance greater than 8,000 µS/cm and a high fraction of water from Malibu Lagoon or seawater (Table S2), was estimated as a three-part mixture of native water, imported water, and seawater (0 per mil). The average percentage of imported water in sampled wells of 30% is similar to the value of 28% reported for recharge to the alluvial deposits in the Civic Center area from OWTS (McDonald Morrissey Associates, Inc. 2010).

Most nitrogen in sampled OWTS was in the form of ammonium, and the δ^15^N-NH_4_ in three of the four sampled residential OWTS was more constant than the chemical composition and ranged from 4.3 to 5.4 per mil (Table S2)—only slightly greater than the average of 4.9 ± 0.4 per mil δ^15^N-NH_4_ reported for human septage (Hinkle et al. 2008; Katz et al. 2010). In contrast, the sample from the OWTS dominated by nitrate rather than ammonium had a δ^15^N-NH_4_ composition of 19 per mil (Table S2). The lower total nitrogen concentration and heavier δ^15^N-NO_3_ composition compared to typical wastewater concentrations (Umari et al. 1993; Wakida and Lerner 2005; Katz et al. 2010) and isotopic compositions (Hinkle et al. 2008) are consistent with nitrogen loss either through volatilization of ammonium or denitrification within this OWTS prior to sample collection. The δ^15^N-NH_4_ in the commercial OWTS was 3.7 per mil (Table S2), which is lighter than the expected composition of human waste, and may reflect nitrogen contributions from food preparation.

δ^15^N-NH_4_ and δ^15^N-NO_3_ in water from sampled wells ranged from 6.1 to 23, and 9.3 to 108 per mil, respectively. These values are heavier than the δ^15^N-NH_4_ values of 4.9 ± 0.4 per mil reported for human waste (Hinkle et al. 2008; Katz et al. 2010) or the δ^15^N-NO_3_ values derived from OWTS discharges of 7.2 ± 2.6 per mil (Hinkle et al. 2008), and reflect isotopic fractionation resulting from chemical and microbiological processes occurring within the groundwater system that transform nitrogen discharged from OWTS.

## Discussion

**Figure 3:**
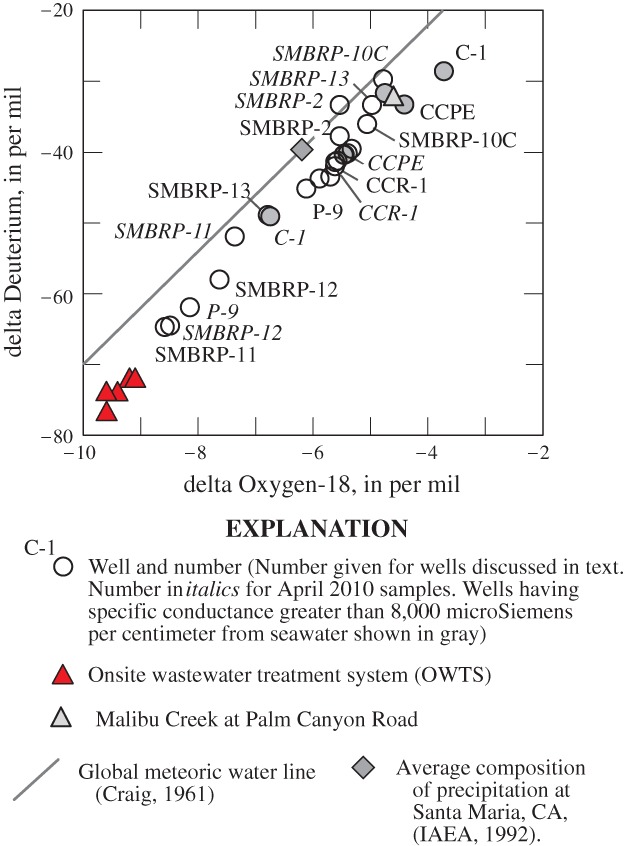
Delta Oxygen-18 (δ^18^O) and delta deuterium (δD) composition of water from wells and from within onsite wastewater treatment systems (OWTS), Malibu, California, July 2009 and April 2010.

In the study area, all water used for public supply, and ultimately discharged from OWTS, is imported from either northern California or from the Colorado River. In the residential and commercial areas of the Civic Center where data were collected, there is very little landscape irrigation and virtually all the imported water recharging the alluvial aquifer has a wastewater history. Discharges from OWTS are often small and in some settings it may be difficult to ensure samples are representative of those discharges. However, δ^18^O and δD values show samples from wells are composed of a wide range of native and imported water, and some wells are predominately composed of water having a wastewater history (Figure 3).

Water from some wells in residential areas near Malibu Colony, such as SMBRP-11 and SMBRP-12, had as much as 70% wastewater (Table 1). High percentages of wastewater also were present in wells P-9 and CCSC-1 in the commercial area during April 2010, although smaller percentages were present in July 2009 (Table 1). In contrast, 39% wastewater was estimated in samples from SMBRP-13 during July 2009 and 23% in April 2010. Seasonal differences in wastewater percentages may occur because of differences in groundwater flow direction (Figure 2) resulting from seasonal differences in lagoon water levels.

In contrast, water from some wells such as SMBRP-10C or SMBRP-2 had little or no wastewater. SMBRP-10C is located in a wetland along the western edge of the Civic Center area in an area removed from Malibu Lagoon and OWTS discharges. SMBRP-2 is located along the eastern edge of Malibu Lagoon also in an area removed from OWTS discharges.

### Processes Affecting Nitrogen

Ammonium and ammonium plus organic nitrogen concentrations in water from sampled wells were positively correlated with increasing estimated percent wastewater, although Spearman Rank Correlation Coefficients of 0.23 and 0.31, respectively, were low. Nitrate was inversely correlated (−0.15) with percent wastewater, suggesting that either ammonium is not being nitrified, or that once nitrified, nitrate from OWTS discharges may be denitrified and lost from the system. δ^15^N-NH_4_ and δ^15^N-NO_3_ data were used to identify processes that control ammonium and nitrate concentrations in groundwater containing water having a wastewater history.

#### Apparent Fractionation

**Figure 4:**
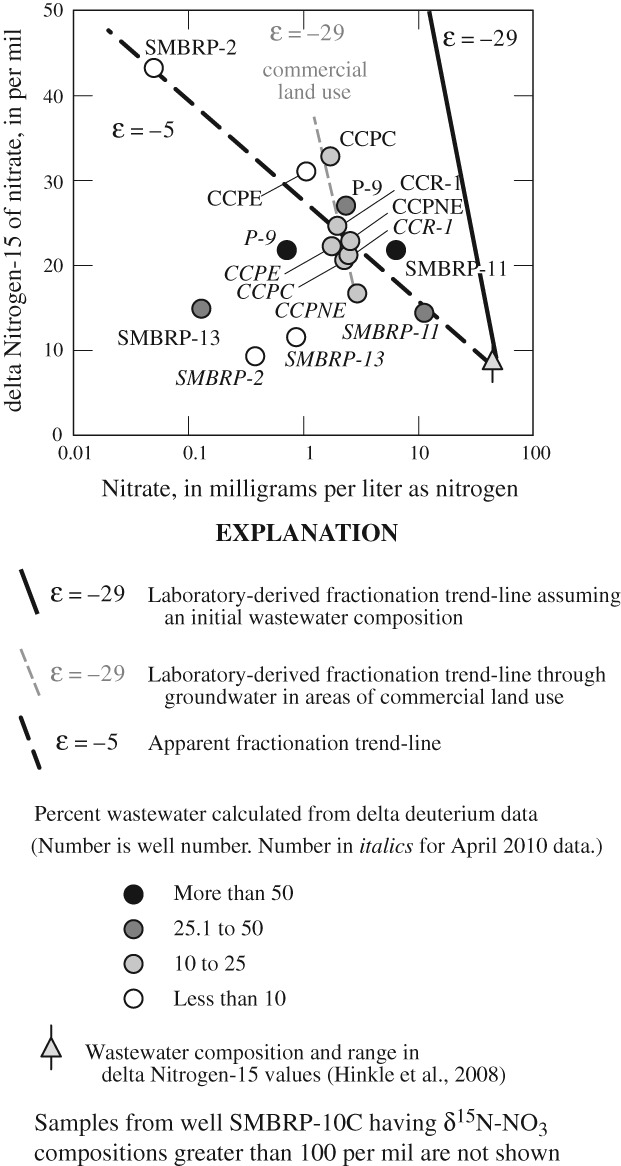
delta Nitrogen-15 of nitrate (δ^15^N-NO_3_) as a function of nitrate concentrations in water from selected wells, Civic Center area, Malibu, CA, July 2009 and April 2010.

The extent of denitrification, believed by previous studies (Suffet and Sheehan 2000; Stone Environmental, Inc. 2004; Sutula et al. 2004) to be the primary nitrate removal mechanism in groundwater underlying the Civic Center area, was initially evaluated using nitrate and δ^15^N isotopic compositions. Nitrate and δ^15^N-NO_3_ were inversely correlated with a Spearman Rank Correlation Coefficient of −0.30, consistent with denitrification (Figure 4).

Presumably, wells having lower nitrate concentrations and heavier δ^15^N-NO_3_ compositions would have undergone more denitrification. However, water from well SMBRP-10C (not shown on Figure 4), located in wetland areas away from unsewered development and having δ^15^N-NO_3_ compositions greater than 100 per mil (Table 1), plots to the far upper left of Figure 4. Similarly, well SMBRP-2 (July 2009) also located away from unsewered development plots to the upper left of Figure 4. Given their locations and the low fraction of wastewater in these samples, the hydrologic history and initial source of nitrogen in water from wells SMBRP-10C and SMBRP-2 (July 2009) must be different from that of most other wells sampled, and water and nitrate in these wells did not originate as discharge from OWTS.

Although there was no consistent pattern in nitrate concentration and δ^15^N-NO_3_ compositions with dissolved oxygen concentrations, or with the fraction of wastewater in the sample; most data fall near a line having an apparent fractionation factor, ε_app_ = −5 (Figure 4). The ε_app_ through these data is within the range reported for ε_app_ for denitrification (Kendall and Aravena 2000), but smaller than the laboratory-derived fractionation factors for denitrification of ε = −29.4 to −24.6 (Mariotti et al. 1981). However, close examination of data from wells near commercial land uses adjacent to Malibu Lagoon (CCPNE, CCPC, CCR-1, CCPE, and P-9) shows data are distributed parallel to the trend calculated from the laboratory-derived fractionation factor for wastewater (Figure 4). Most of these samples are reducing with dissolved oxygen concentrations less than 0.5 mg/L (Table S1), and are in an environment where nitrate reduction may occur. In contrast, samples from residential land uses (SMBRP-13 and SMBRP-11) plot below and to the left of the apparent fraction trend line (ε_app_ = −5) and appear to have little change in δ^15^N-NO_3_ composition with changing nitrate concentration (Figure 4). These samples are oxic with dissolved oxygen concentrations greater than 1 mg/L (Table S1), and are in an environment where nitrate reduction would not occur. SMBRP-2 (April 2010) also plots below and to the left of the apparent fractionation trend line.

Nitrate and δ^15^N-NO_3_ data do not provide a complete understanding of nitrate sources, mixing, denitrification, and other processes that control nitrate concentrations in the Civic Center area. Total nitrogen concentrations and δ^15^N-(NH_4_ + NO_3_) isotopic compositions were evaluated to address the combined effects of these processes on nitrogen concentrations in the Civic Center area.

#### Mixing, Sorption, and Volatilization of Ammonium

**Figure 5:**
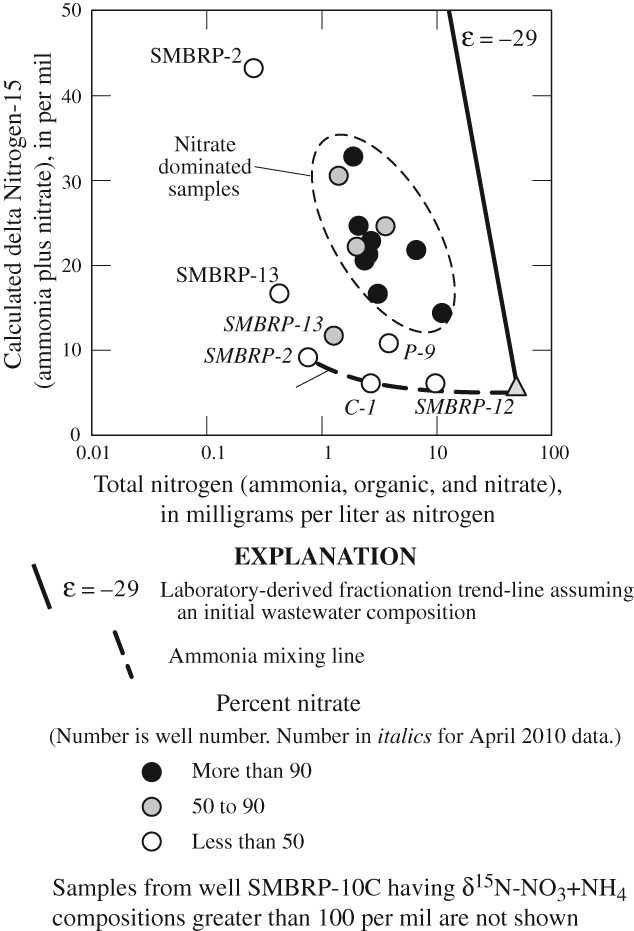
Delta Nitrogen-15 of nitrate plus ammonia (δ^15^N-(NH_4_+NO_3_)) as a function of total nitrogen concentrations in water from selected wells, Civic Center area, Malibu, California, July 2009 and April 2010.

In parts of the study area where groundwater did not contain oxygen, ammonium concentrations would initially be controlled by mixing with native groundwater—although losses of ammonium could occur through sorption onto aquifer deposits, or through volatilization. Total nitrogen concentrations and δ^15^N-(NH_4_ + NO_3_) isotopic compositions for samples dominated by ammonium (SMBRP-12 and C-1, April 2010) plot along a mixing line between OWTS sources and native groundwater (SMBRP-2, April 2010) (Figure 5). Assuming an initial total nitrogen concentration in OWTS discharges of 45 mg/L as N (Umari et al. 1993; Wakida and Lerner 2005; Katz et al. 2010), the concentrations expected from dilution with native groundwater were calculated from the percentage of wastewater estimated on the basis of δ^18^O and δD data. The difference between the measured and expected concentrations was interpreted as nitrogen removal through sorption or volatilization of ammonium. The data suggest that as much as 30 to 50% of the ammonium from OWTS may have been removed through sorption or volatilization. Although imprecise because nitrogen concentrations in OWTS discharges are variable, the data are consistent with significant removal of nitrogen from OWTS discharges without nitrification and subsequent denitrification.

Sorption does not result in isotopic fractionation, and the lack of a shift in the δ^15^N-(NH_4_ + NO_3_) isotopic composition with decreasing nitrogen concentration in *SMBRP-12* and *C-1*, suggests that sorption is the primary removal mechanism for ammonium in water from these wells. In contrast, shifts toward heavier δ^15^N values in water from wells P-9, SMBRP-13 (both July 2009 and April 2010) and SMBRP-2 (July 2009) are consistent with a combination of mixing, sorption, and volatilization of isotopically lighter ammonium. Because the shifts in the δ^15^N-(NH_4_ + NO_3_) are small, sorptive losses of ammonium are probably the dominate nitrogen removal mechanism in water from these wells. Volatilization losses estimated on the basis of 1) literature derived fractionation factors ranging from −13 to −38 (Delwiche and Steyn 1970; Mariotti et al. 1981; Casciotti et al. 2003), and 2) the difference in δ^15^N-NH_4_ compositions in wells P-9, SMBRP-13 (both July 2009 and April 2010) and SMBRP-2 (July 2009) and the average δ^15^N-NH_4_ composition of septage (Hinkle et al. 2008; Katz et al. 2010) range from less than 5% to as high as 35% of the initial ammonium concentration. However, ammonium losses through volatilization do not exceed 10% in most of these samples.

Removal of ammonium through sorption or volatilization, without nitrification occurred primarily in April 2010 (Figure 5). This may be because cooler temperatures may inhibit conversion of ammonium to nitrate compared to warmer temperatures in July 2009.

#### Nitrification and Denitrification

Nitrification can occur either in the unsaturated zone or in shallow groundwater. Samples that contain nitrate also may have undergone some combination of mixing with native groundwater and losses of ammonium through sorption or volatilization prior to nitrification, and subsequent denitrification. The order in which these reactions occur, and the extent of these reactions, were evaluated on the basis of the δ^18^O-NO_3_ composition.

**Figure 6:**
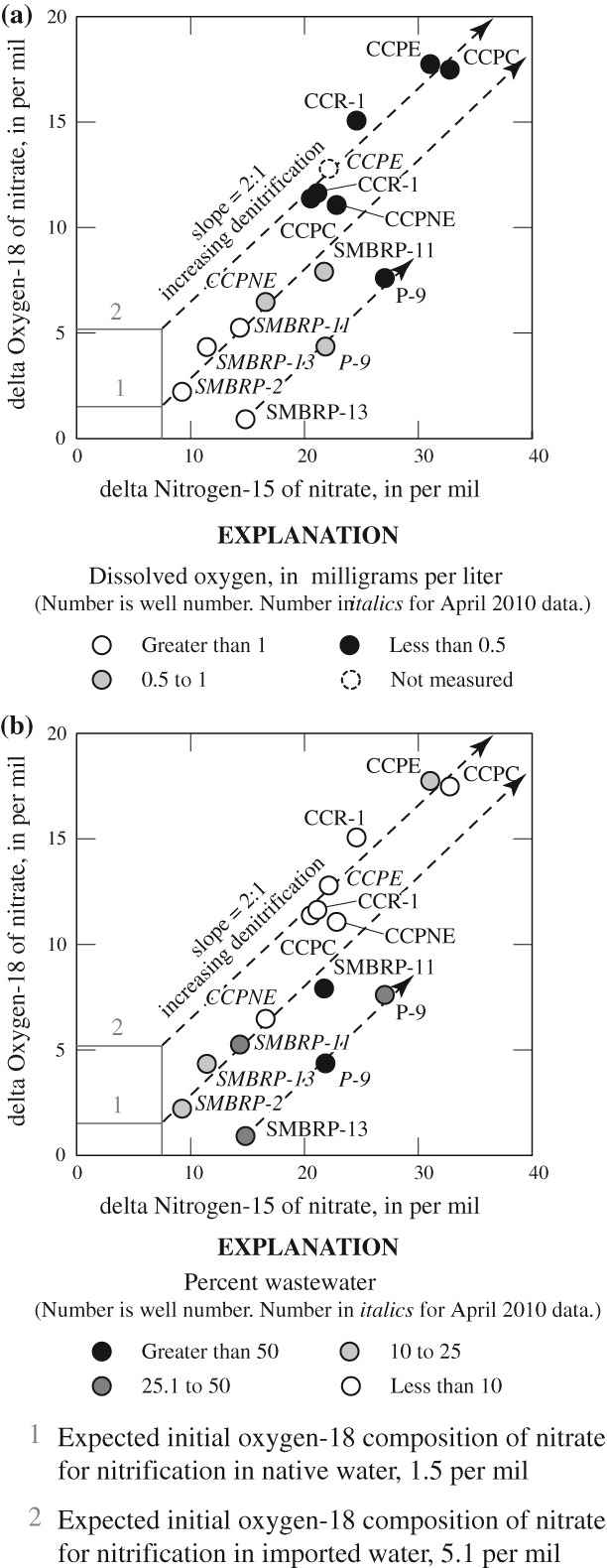
Delta Oxygen-18 of nitrate (δ^18^O-NO_3_) as a function of delta Nitrogen-15 of nitrate (δ^15^N-NO_3_) in water from selected wells, (a) with respect to dissolved oxygen concentrations and (b) with respect to percent wastewater, Civic Center area, Malibu, California, July 2009 and April 2010.

During nitrification, one-third of the oxygen in nitrate is derived from dissolved oxygen in the water (having a δ^18^O composition of about 23 per mil), and two-thirds of the oxygen is derived from oxygen in the water molecule through hydrolysis (Mayer et al. 2001). Given an initial δ^15^N-NO_3_ composition of nitrate in OWTS discharges derived from human waste of about 7 per mil (Hinkle et al. 2008); for samples where the water was composed of a large percentage of imported water (having a δ^18^O composition of about −9.5 per mil), the initial composition of the oxygen in the nitrate formed during nitrification would be about 1.5 per mil (Figure 6). For samples where extensive mixing of native water (having a δ^18^O composition of about −4 per mil) and imported water occurred prior to nitrification, the initial composition of the oxygen in nitrate nitrified from ammonium in the study area could be as heavy as 5.1 per mil (Figure 6). As denitrification occurs, the δ^15^N-NO_3_ and δ^18^O-NO_3_ compositions change in a predictable fashion as a result of isotopic fractionation along a line having a slope of 2 (Figure 6). More denitrification and removal of nitrogen has occurred to the right along these lines; less denitrification and removal of nitrogen has occurred to the left.

In general, samples from wells near commercial land uses (CCPC, CCPE, CCR-1, and CCPNE) have under-gone more denitrification than samples from wells near residential land uses (SMBRP-11 and SMBRP-13) (Figure 6). On the basis of shifts in the δ^15^N-NO_3_ composition along the ε = −29 trend line through samples in the commercial area (Figure 4), nitrate removal through denitrification in this area ranges from 40 to 60% of the nitrate from OWTS discharges, with some additional prior loss of nitrogen through volatilization of ammonium, or loss of nitrous oxides intermediaries. Denitrification is consistent with low dissolved oxygen concentrations in groundwater in the commercial area, and the denitrification trend line intercepts the expected δ^15^N-NO_3_ composition from wastewater at a δ^18^O-NO_3_ composition consistent with nitrification occurring in native water (Figure 6). As a consequence, it is likely that nitrification occurred in the saturated zone after mixing of OWTS discharges and native groundwater. In contrast in the residential area, nitrate removal rates range from less than 5 to 40% of the nitrate from OWTS discharges, with some additional prior loss of nitrogen through volatilization of ammonium or loss of nitrous oxide intermediaries. Less denitrification in the residential area is consistent with more oxic groundwater. In the residential area, the denitrification trend line intercepts the expected δ^15^N-NO_3_ composition from wastewater at a δ^18^O-NO_3_ composition consistent with nitrification occurring prior to mixing of OWTS discharges and native groundwater (Figure 6). As a consequence, it is likely that nitrification occurred in the unsaturated zone prior to mixing with native groundwater. Irrespective of land use, denitrification was more extensive during the July 2009 sample period than during the April 2010 sample period, probably because of warmer temperatures and higher groundwater levels during the summer months.

In contrast to water from most other wells, the dentrification trend line through wells P-9 and SMBRP-13 (July 2009) intercept expected δ^18^O-NO_3_ compositions at a values near −6 per mil. This may be the result of the incomplete nitrification of ammonium to nitrate in these wells with the initially isotopically lighter δ^15^N-NH_4_ preferentially nitrified. The calculated δ^15^N-(NH_4_ + NO_3_) composition of water from these wells (Table S2) is different from the expected δ^15^N-NH_4_ composition from human waste discharged from OWTS and is consistent with significant nitrogen losses either through volatilization of ammonium or intermediary nitrous oxides during nitrification. Consistent with previous data, the δ^15^N-NO_3_ and δ^18^O-NO_3_ from well SMBRP-10C and the δ^15^N-NO_3_ values from well SMBRP-2 (July 2009) are heavier than other samples (Table S2). Water from these wells do not contain OWTS discharges (Table 1) and these values may reflect nitrogen sources and a hydrologic history different from other samples.

#### Nitrogen Removal

Nitrogen removal from groundwater in the Civic Center area is dependent on the form of the nitrogen. Sorption and to a lesser extent volatilization of ammonium can contribute to as much as 30 to 50% and 10% removal of nitrogen from OTWS discharges, respectively. Subsequent denitrification can remove as much as 40 to 60% of the remaining nitrogen from groundwater in the commercial area and 5 to 20% of the nitrogen from groundwater in the residential area. Nitrogen removal through sorption dominated during the cooler April sample period and nitrogen removal through denitrification dominated during the warmer July sample period.

Although median nitrogen concentrations are within ranges that suggest nitrogen may contribute to eutrophic conditions in surface water (Dodds et al. 1998), aquifer deposits become progressively finer-grained and more reducing with proximity to Malibu Lagoon—possibly contributing to increased removal of nitrogen through sorption of ammonium and through denitrification of nitrate. The data suggests that nitrogen in groundwater may be a smaller contributor to eutrophic conditions in the Malibu Lagoon than suggested in previous studies (Ambrose and Orme 2000; Sutula et al. 2004). It is possible that nitrogen in the lagoon may be derived from a combination of recycling of nitrogen in fine-grained sediments on the lagoon bottom, fertilization by birds, or from fixation of nitrogen by algae within the lagoon.

### Processes Affecting Phosphate

Phosphate concentrations in native groundwater, unaffected by wastewater discharges or agricultural activities tend to be low, typically about 0.02 mg/L (Fuhrer et al. 1999). Phosphate has long been believed to be relatively immobile in groundwater as a result of mineralogic controls and sorption of phosphate to iron oxides on mineral surfaces (Hem 1985; Mueller and Helsel 1996).

The possibility of mineralogic controls on phosphate concentrations in the study area was evaluated with respect to solubility of hydroxyapatite, Ca_10_(PO_4_)_6_(OH)_2_, on the basis of saturation indexes calculated using the computer program WATEQ4F (Ball and Nordstrom 1991). When using this approach, saturation indexes less than 0 are undersaturated with respect to the mineral of interest, and if present the mineral would tend to dissolve. Saturation indexes greater than 0 are supersaturated with respect to the mineral of interest and, in the absence of kinetic controls the mineral would tend to precipitate. If a mineral is dissolving or precipitating at rates sufficient to influence the chemistry of the water, the saturation index should be near 0, typically ± 0.5.

**Figure 7:**
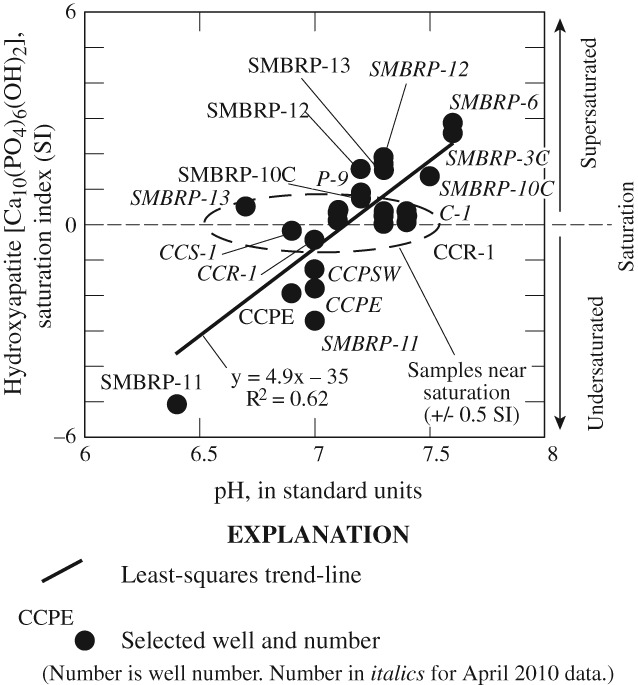
Hydroxyapatite saturation as a function of pH in water from wells in the Civic Center area, Malibu, California, July 2009 and April 2010.

Almost half of the groundwater samples from the Civic Center area have saturation indexes within ± 0.5 of saturation for hydroxyapatite (Figure 7). Phosphate concentrations in these samples ranged from 0.12 to 2.2 mg/L, and mineral solubility (with respect to hydroxyapatite) does not keep phosphate in groundwater below concentrations that limit eutrophication in surface waters receiving groundwater discharge. Hydroxyapatite solubility is a function of pH with undersaturation at lower, slightly acidic, pHs and oversaturation at higher more alkaline pHs (Figure 7). pH was slightly inversely correlated with the fraction of wastewater sample (*r* = −0.17), possibly as a result of decreases in pH associated with the bacterial respiration and production of CO_2_ from organic material in the OWTS discharges. Hydroxyapatite solubility was not correlated with the fraction of wastewater in a sample, but was correlated with ammonium (*r* = 0.56) possibly because of more acidic conditions associated with NH_4_^+^ in solution.

**Figure 8:**
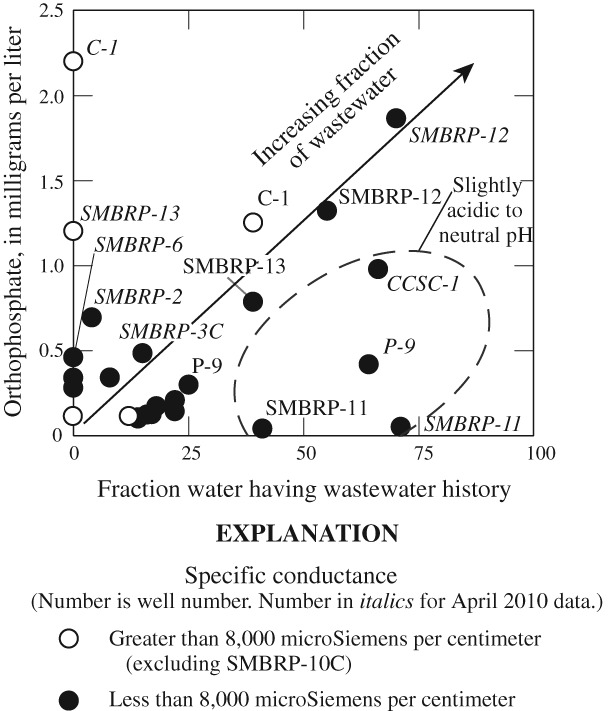
Orthophosphate concentrations as a function of increasing percentage of imported water having a wastewater history in water from wells in the Civic Center area, Malibu, California, July 2009 and April 2010.

In contrast to mineral solubility, sorption to iron oxides on the surfaces of mineral grains may limit phosphate concentrations in groundwater (Hemwell 1957; Mueller and Helsel 1996; Zhang and Huang 2007). Sorption of phosphate is pH dependent, and more effective at slightly acidic to neutral pH. For groundwater having slightly alkaline pH (greater than pH 7.3) in the Civic Center area, phosphate concentrations increase linearly along a mixing line with increasing fraction of wastewater (Figure 8). In contrast, groundwater having a high percentage of wastewater and neutral to slightly acid pH plots below the mixing line, consistent with pH dependent sorption of phosphate by iron oxides (Domagalski and Johnson 2011).

## Conclusions

Recharge to the alluvial aquifer underlying the Malibu Civic Center from OWTS discharges is about 28% of the total recharge. On the basis of δ^18^O and δD data, water from wells sampled as part of this study contained on average 30% imported water having a wastewater history—water from some sampled wells contained more than 70% wastewater from OWTS discharges.

Ammonium was the dominant form of nitrogen in 23% of samples from wells in the Malibu Civic Center area, and was the dominant form of nitrogen for samples subjected solely to reduced conditions. In reduced conditions, sorption of ammonium was the primary mechanism for nitrogen removal, with removal as high as 30 to 50% of the total nitrogen released by OWTS discharges. Volatilization of ammonium removed smaller amounts of ammonium, commonly less than 10% of the total nitrogen, released by OWTS. Volatilization of ammonium varies seasonally, with less volatilization occurring during the cooler winter months and more volatilization occurring during the warmer summer months.

Nitrate was the dominant form of nitrogen in 58% of samples from wells, and was the dominant form of nitrogen for samples exposed to oxic conditions after discharge from OWTS. After ammonium was converted to nitrate, subsequent exposure to reduced conditions within the aquifer promoted removal of nitrogen by denitrification. In the commercial area, nitrate removal through denitrification ranged from 40 to 60% of the total nitrogen in OWTS discharges. In the residential area, where groundwater was more oxic, nitrate removal through denitrification was less than 20% of the total nitrogen.

Nitrogen removal varied with environmental factors (primarily redox conditions, related to the presence or absence of dissolved oxygen), and with land use (probably a function of greater OWTS discharges in commercial compared to residential land use). Although previous studies identified seasonal differences in nitrogen removal, they did not distinguish between different processes that act to remove nitrogen in the study area. Removal of up to 30 to 50% of ammonium through sorption, and removal of up to 40 to 60% of nitrate through denitrification near commercial land uses are within overall ranges for nitrogen removal estimated by Stone Environmental, Inc. (2004). However, additional nitrogen removal is likely farther downgradient along groundwater flow paths, especially in fine-grained, organic-rich sediments near Malibu Lagoon. As a consequence, it is possible that almost complete removal of nitrogen may occur before groundwater discharges to Malibu Lagoon.

Initial examination of the nitrate and δ^15^N-NO_3_ data yielded apparent fraction factors (ε_app_) within reported ranges (Kendall and Aravena 2000). Independent estimation of the fraction of wastewater present in a sample on the basis of δ^18^O and δD data, coupled with the lack of confounding nitrogen inputs to groundwater, permitted separate examination of (1) dilution from mixing with native groundwater and (2) individual processes contributing to nitrogen removal. In this study, nitrogen removal by dentrification and other processes could be quantified using laboratory-derived fractionation factors (ε). Results of this study show that aquifer heterogeneity and mixing of waters from different sources, although probably important (Abe and Hunkeler 2006; Green et al. 2010), are not the only cause of differences between apparent fraction factors estimated from field data and laboratory derived fractionation factors. Future studies of the effect of aquifer heterogeneity on geochemical processes and isotopic fractionation may benefit from careful processes-oriented isotopic work prior to analytical or numerical modeling.

Unlike nitrogen, there are fewer processes in the study area that control phosphate concentrations from OWTS discharges in groundwater—although pH dependent sorption of phosphate to iron and manganese oxides on the surfaces of mineral grains at slightly acidic to near-neutral pH may remove some phosphate in the study area. Groundwater discharge containing phosphate from OWTS discharges are sufficiently high to contribute to eutrophic conditions in sensitive receiving waters such as Malibu Lagoon.

## Supporting Information

Table S1 Field Parameters, Nitrogen and Phosphorus Concentrations in Water from Sampled Wells and Within Onsite Wastewater Treatment Systems (OWTS), Civic Center area, Malibu, California, July 2009 and April, 2010.

Table S2 Isotopic Composition of Water, Ammonium, and Nitrate from Sampled Wells and Within Onsite Wastewater Treatment Systems (OWTS), Malibu, California, July 2009 and April 2010.
